# CD4-cell counts and presence of AIDS in HIV-positive patients entering specialized care—a comparison of migrant groups in the German ClinSurv HIV Cohort Study, 1999–2013

**DOI:** 10.1186/s12879-016-2070-5

**Published:** 2016-12-07

**Authors:** Nadine Zeitlmann, Barbara Gunsenheimer-Bartmeyer, Claudia Santos-Hövener, Christian Kollan, Matthias an der Heiden, On behalf of the ClinSurv Study Group, K. Arastéh, D. Hampf, F. Bergmann, M. Warncke, J. Rockstroh, J. Wasmuth, S. Hass, B.-E. O. Jensen, L. Rollmann, S. Esser, P. Schenk-Westkamp, A. Haberl, C. Stephan, A. Plettenberg, F. Kuhlendahl, A. Adam, L. Weitner, K. Schewe, H. Goey, S. Fenske, T. Buhk, H. J. Stellbrink, C. Hoffmann, J. van Lunzen, A. Zoufaly, K. Wassmus, M. Stoll, S. Gerschmann, H. Horst, S. Trautmann, G. Fätkenheuer, D. Gillor, J. Bogner, B. Sonntag, B. Salzberger, C. Fritzsche

**Affiliations:** 1 Postgraduate Training for Applied Epidemiology (PAE), Robert Koch Institute, Berlin, Germany; 2 European Programme for Intervention Epidemiology Training, ECDC, Stockholm, Sweden; 3 Department for Infectious Disease Epidemiology, Robert Koch Institute, Berlin, Germany; 4 Department for Infectious Disease Epidemiology, Bavarian Health and Food Safety Authority, Oberschleissheim, Germany

**Keywords:** HIV, Care, Migrants, Germany, CD4, AIDS, Sub-Saharan Africa, South-East Asia, Late presentation

## Abstract

**Background:**

Although early presentation to HIV-care is essential to ensure timely initiation of antiretroviral therapy, recent studies have shown that especially migrants present to HIV-care at a later stage of HIV-infection. Currently, thirty percent of all newly diagnosed HIV cases in Germany originate from abroad. So far it is unknown, which specific migrant groups in Germany are particularly at risk for late presentation to HIV-care.

**Methods:**

We used data from the Clinical Surveillance of HIV Disease (ClinSurv) cohort, a multi-centre observational cohort (01/01/1999 and 31/07/2013) and included treatment-naïve patients with valid information on country of origin and date of enrolment. Migrants were patients with country of origin outside Germany. We compared time trends for percentage of AIDS (CDC Stage C) and mean CD4-count at enrolment between migrants from Western Europe (WE), Central Europe (CE), Eastern Europe (EE), Sub-Saharan Africa (SSA), South East Asia (SEA) and non-migrants using multivariable regressions. Male non-migrants with mean age of 38-years constituted the reference group.

**Results:**

In total, 10,211 patients fulfilled the inclusion criteria, of which 2784 were migrants (SSA: 42%, CE: 17%, WE: 11%, EE: 10%, SEA: 9%). The percentage of patients with AIDS at enrolment was higher in SSA (Odds Ratio (OR)_SSA_: 1.44, 95%-confidence interval (95%-CI):1.12–1.84) and SEA-migrants (OR_SEA_:2.16, 95%-CI:1.43–3.27). In addition, female SEA-migrants, were more likely to present with AIDS than their male counterparts (OR:2.22, 95%-CI:1.18–4.17). Mean CD4-count at enrolment was lower for SSA- (Mean CD4-count ratio (IRR):0.72; 95%-CI:0.64-0.82) and SEA-migrants (IRR:0.62, 95%-CI:0.49-0.78). Over time, it increased in non-migrants and CE-migrants (by 1 and 3%/year, respectively), whereas no increase was seen for SEA and SSA.

**Conclusions:**

SSA and SEA-migrants in Germany present to HIV-care at a later stage of HIV infection than non-migrants. Additionally, previous research found a higher risk for late HIV-testing for migrants. Collecting information about the arrival date of migrants in Germany in the HIV notification system would help to understand to which extent these problems could be tackled in Germany. Moreover, participatory approaches for HIV-testing and care as well as research regarding knowledge, behaviour and attitudes towards these topics for SSA and SEA migrants should be expanded.

## Background

According to the latest International Migration Report of the United Nations, there are over 200 million migrants worldwide, of which 10 million reside in Germany [1].

In Europe between 2007 and 2012, 40% of the cases of Human Immunodeficiency Virus (HIV) reported to the European Centre for Disease Prevention and Control (ECDC), originated from a country outside the country where their HIV infection was diagnosed [2]. In both reports migrants are defined as foreign-born or, when country of birth was not available, foreign citizens. In the German notification data on new HIV-diagnoses information on country of birth or citizenship is not collected. Hence migrants are defined by country of origin, namely as cases, which have spent the majority of their life time outside Germany. From 2002 to 2013, around 30% of new HIV-diagnoses in Germany are seen in migrants [3].

Generally, HIV-testing in Germany is offered free-of-charge. In addition, anonymous testing is possible at most local public health voluntary counselling and testing sites and at several non-governmental organizations. HIV-testing for pregnant women and patients with suspected HIV-infection at the general practitioners and specialists is covered by statutory health insurances [4].

German-Austrian antiretroviral treatment (ART) guidelines for HIV-infection for adults recommended that ART should be initiated in all symptomatic patients especially after the onset of at least one illness defining acquired immunodeficiency syndrome (AIDS) [5, 6]. Furthermore, they also consider CD4 T-cell counts of below 350 CD4 cells/μl as a threshold for treatment indication, since ART was found more beneficial regarding a decelerated progression to AIDS and death when initiated at higher CD4-counts [7, 8]. Currently, worldwide HIV research societies discuss an update of national HIV-treatment guidelines to recommend ART-initiation irrespective of CD4-cell count. This is a consequence of recently published results of the Strategic Timing for Anti-Retroviral Treatment (START) Trial showing benefits regarding HIV-outcomes in patients timing ART-initiation at CD4-counts >350 cells/μl over patients with deferred treatment initiation [9, 10].

ART in Germany is offered to all HIV-positive patients. It is fully paid by statutory health insurance, which postulates treatment by specialized physicians [11]. In order to enable timely initiation of ART according to guidelines, an early presentation of HIV-positive patients to HIV-care is essential.

Past studies on clinical features in migrants in Germany have already shown an association of migration status with late presentation for HIV-care [12]. However, the present study aims to analyse late presentation to HIV-care with regards to different groups of migrants. In this context, we compared the percentage of patients presenting with AIDS, as well as the CD4-cell count at entry to specialized care among different migrant populations and non-migrants in a long term cohort study of HIV positive patients in Germany.

## Methods

### Data source

We used data from the Clinical Surveillance of HIV Disease (ClinSurv) cohort. This open multi-centre long term observational cohort was established on 1^st^ January 1999 and is a network of 15 HIV treatment centres and university clinics in major German cities, which collect data on about 19.000 HIV-positive patients (as of 31 July 2013) with unknown date of HIV-infection. For each patient who seeks care in a participating centre, information on age, sex, country of origin (collected by the treating physician according to patient’s information) and transmission group category was reported at enrolment in ClinSurv. Clinical parameters (such as occurrence of opportunistic diseases, Centers for Disease Control and Prevention (CDC) stage, immunological and virological status (viral load and CD4 T-cell count) of HIV infection as well as information on ART (date of initiation, combination of drug classes in therapy regimes, therapy interruption) are recorded upon enrolment and updated for every date in which the patient visits the centre after enrolment. The collected data is sent biannually in an encrypted format to the Robert Koch Institute, where it undergoes verification and quality checks [13]. The Robert Koch Institute is the German national public health institute; therefore the Federal Commissioner for Data Protection is the responsible entity for studies conducted by the Robert Koch Institute. Information on HIV infection collected in ClinSurv corresponds to the data reported to the RKI according to legal requirements implemented by the national Protection against Infection Act (IfSG) of 2001. All patient data collected in ClinSurv are generated during routine care. The German Federal Commissioner for Data Protection therefore waived the need for ethical approval for the ClinSurv study. No written informed consent is required from patients.

The ClinSurv cohort collects anonymized data on all HIV-patients, who are in medical care in these 15 participating centres.

Authors from the Robert Koch Institute, as the data processing site, only had access to anonymized data and had no access to identifying information. All data analyses were performed on the anonymized data set. There were no permissions required to conduct scientific analyses.

For our analysis we extracted all entries of patients enrolled in ClinSurv between 1 January 1999 and 31 July 2013, who were older than 15 years and treatment naïve at enrolment in ClinSurv and where the definite time of enrolment into ClinSurv was known. Furthermore, only patients, where sex and country of origin were available in the database were included. We excluded patients who acquired HIV-infection through haemophilia, mother-to-child-transmission or by occupational exposure (rare transmission routes).

### Definitions

As described above, a migrant was defined as a patient with a reported country of origin outside Germany. Migrants were further categorized in migrant groups by regions of origin according to the World Health Organization (WHO) region definitions [14].

AIDS was defined by CDC Stage C diagnosis. Here the date of diagnosis before enrolment or within 45 days after enrolment was classified as presenting with AIDS at enrolment into the cohort. We obtained the CD4-count in cells/μl of a patient when presenting to specialized care by using the CD4-count measured at the enrolment of the patient into ClinSurv.

### Statistical analysis

In order to get an overview of the study population, we analysed the most frequent regions of origin for migrants descriptively. For non-migrants and migrants by region of origin, the median age, percentage of males, most frequent risk groups and mean CD4-counts and proportion of patients with AIDS were compared. We performed rank sum and Chi-squared tests to test for differences in sex and age at ART-initiation between migrants overall and non-migrants.

We performed multivariable regressions to estimate the difference in CD4-count and percentage of patients with AIDS prior to enrolment into ClinSurv between non-migrants and the five most frequent migrant groups. The other migrant regions, which were represented by less than 200 enrolled patients over the timespan 1999 to 2013, were excluded from the regression models. Instead of the continuous age variable used in the descriptive analysis, for the regression analysis, age groups (in 10 year intervals) were formed in order to consider also non-linear effects of age. Regarding CD4-count we performed negative binomial regressions including only patients with available information on CD4-count at their enrolment visit. The obtained incidence rate ratios (IRR) in our model refer to the mean CD4 count ratio. IRR were calculated with their 95%-confidence intervals (95%-CI). In the negative binomial regression, the variance can be estimated through the dispersion parameter. To compare the percentage of patients with AIDS, odds ratios (OR) and their 95%-CI were analysed in logistic regressions. In addition a time-variable was included in the regression models, to describe the trends of these values over the time period 01/01/1999 to 31/07/2013. Male non-migrants in the age group 35–44 years (which included the median age of this subgroup) constituted the reference group. An alpha of 0.05 was considered the level indicating statistical significance.

All analyses were performed with the software STATA 14.0.

## Results

**Fig. 1:**
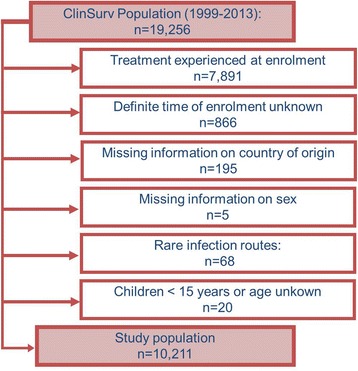
Study population and excluded patients from ClinSurv

**Fig. 2:**
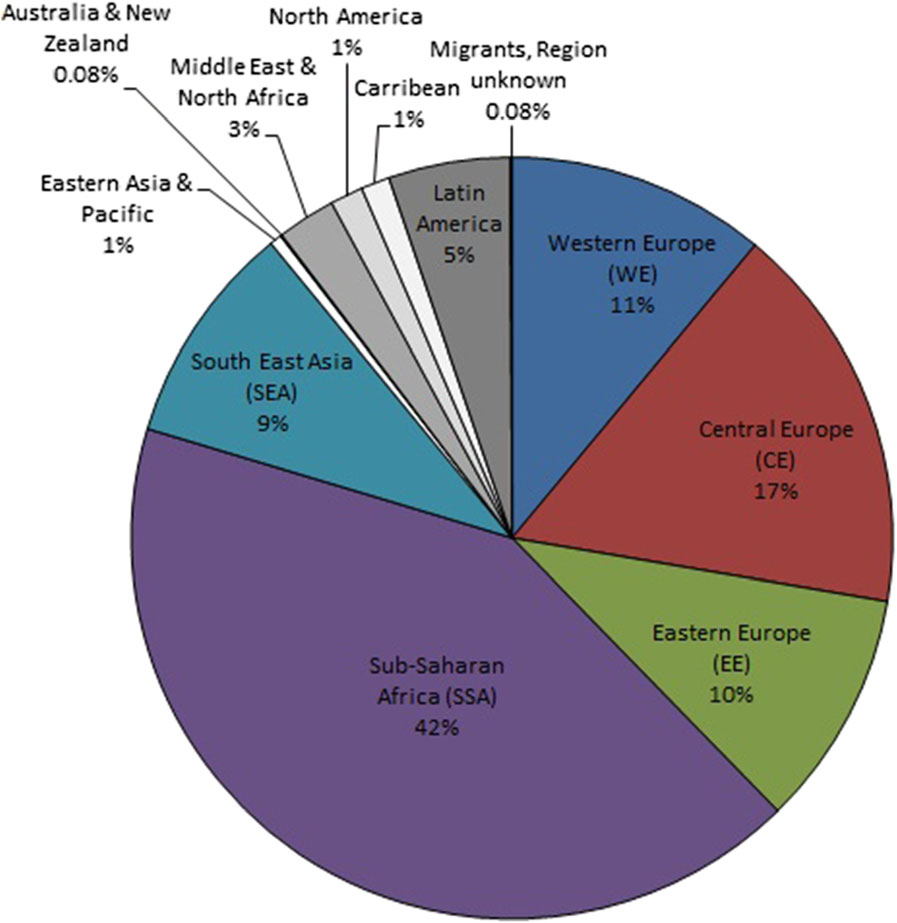
Region of origins of migrants (*n* = 2784) in the study population, 1999–2013

Of 19,256 patients enrolled in the ClinSurv cohort as of 31/07/2013, 10,211 patients fulfilled the inclusion criteria (see Fig. 1 for details). Of those, 2784 (27%) were migrants. The majority of migrants (42%) originated from Sub-Saharan Africa (SSA), followed by Central Europe (CE, 17%), Western Europe (WE, 11%), Eastern Europe (EE, 10%) and South-East Asia (SEA, 9%). Rare regions of origin were amongst others Latin America, and the Middle East and North-Africa Region with 5 and 3%, respectively (see Fig. 2).

Migrants overall enrolled in the cohort at a significantly younger age (median age: 34 years) than non-migrants (median age: 38 years) (*p*-value: <0.001) and were less often male (60% versus 88%, respectively) (*p*-value: <0.001). While Western and Central Europeans show a more similar distribution of these characteristics to non-migrants, migrants from Eastern Europe display the lowest age at enrolment. Migrants from Sub-Saharan Africa are least frequently male (39%).

Non-migrants, Western Europeans and migrants from Central Europe acquired HIV-infection predominantly via the Men-who-have-sex-with-men (MSM)-transmission route (64%, 61% and 45%, respectively). For migrants from Eastern Europe, Injecting drug use (IDU) as a transmission route was noticeably higher (27%) compared to other migrant regions (see Table 1).

**Table 1:** Characteristics of the study population at enrolment, 1999–2013, (*n* = 9320; only selected migrant groups shown)

Characteristic at enrolment	Non-Migrants	Western Europe	Central Europe	Eastern Europe	Sub-Sahara Africa	South-East Asia
Total number of patients	7,427	307	463	280	1,167	263
Median Age (in years); (interquartile range)	38 (31–45)	37 (31–44)	34 (29–42)	31 (26–37)	33 (28–39)	33 (28–39)
Sex						
Female; n (%)	890 (12)	33 (11)	85 (18)	110 (39)	708 (61)	127 (48)
Male; n (%)	6537 (88)	274 (89)	378 (82)	170 (61)	459 (39)	136 (52)
Transmission Group; n (%)						
MSM	4,724 (64)	187 (61)	209 (45)	62 (22)	22 (2)	72 (27)
IDU	610 (8)	29 (9)	41 (9)	76 (27)	7 (0.60)	6 (2)
Hetero	1,348 (18)	50 (16)	151 (33)	94 (34)	3 (0.26)	31 (12)
High prevalence country (HPC)^a^	--	7 (2)	5 (1)	6 (2)	1,135 (97)	133 (51)
Unknown	745 (10)	34 (11)	57 (12)	42 (15)	--	21 (8)
Patients with AIDS; n (%)	1,086 (15)	43 (14)	66 (14)	18 (6)	184 (16)	72 (27)
Median CD4-count (cells/μl); (interquartile range)	340; (162–520)	305; (122–521)	310; (140–467)	398; (235–561)	253; (120–410)	198; (64–369)

^a^Transmission group HPC (High prevalence country): origin from a high prevalence country for HIV-infection, when other transmission group category is unknown. A high prevalence country is defined as a country with an adult HIV infection prevalence of >1% [14]

### AIDS

In non-migrants and migrants from Western Europe, Central Europe and Sub-Saharan Africa approximately 15% of patients join ClinSurv after AIDS is present. For migrants from South-East Asia this percentage is elevated (27%) (see Table 1). In the multivariable analysis, among male patients, migrants from Sub-Saharan Africa and South-East Asia were more likely to enrol after diagnosis of AIDS compared to non-migrants (OR_SEA_: 2.16, 95%-CI:1.43 – 3.27; OR_SSA_: 1.44, 95%-CI: 1.12 – 1.84). Overall no difference in AIDS at enrolment between male and female patients was found, except for South East Asian migrants, where females are more likely to present with AIDS to specialized care than their male counterparts (OR = 2.22, 95%-CI:1.18–4.17) (see Table 2).

**Table 2:** Multivariable logistic regression model for time trend of percentage with AIDS at enrolment, 1999–2013 (*n* = 9907); significant results in bold

Percentage with AIDS prior to enrolment	OR	95%-CI	*p*-value
**Region of origin of migrant group**			
Non-migrants	Ref.	--	--
WE	0.90	0.70–1.39	0.952
CE	1.20	0.90–1.60	0.222
**EE**	**0.52**	**0.30–0.96**	**0.038**
**SSA**	**1.44**	**1.12–1.84**	**0.004**
**SEA**	**2.16**	**1.43–3.27**	**<0.001**
**Sex**			
Male	Ref.	--	
Female	1.17	0.81–1.67	0.403
**Sex and Region of origin, (2013)**			
Female WE	0.55	0.12–2.43	0.428
Female CE	0.49	0.20–1.21	0.122
Female EE	1.39	0.50–3.83	0.526
Female SSA	1.02	0.69–1.51	0.916
**Female SEA**	**1.90**	**1.05–3.46**	**0.035**
**Age Group (in years)**			
**15–24**	**0.31**	**0.23–0.42**	**<0.001**
**25–34**	**0.67**	**0.58–0.78**	**<0.001**
**35–44**	**Ref.**	**--**	**--**
**45–54**	**1.57**	**1.34–1.83**	**<0.001**
**55+**	**1.90**	**1.57–2.30**	**<0.001**
**Time trend (per year) by sex; 1999–2013**			
Female	0.99	0.96–1.03	0.742
**Male**	**0.94**	**0.93–0.96**	**<0.001**
**Percentage with AIDS prior to enrolment**	**Odds**	**95%-CI**	***p*** **-value**
**(reference group, 2013)**	**0.12**	**0.10–0.14**	**<0.001**

**Fig. 3:**
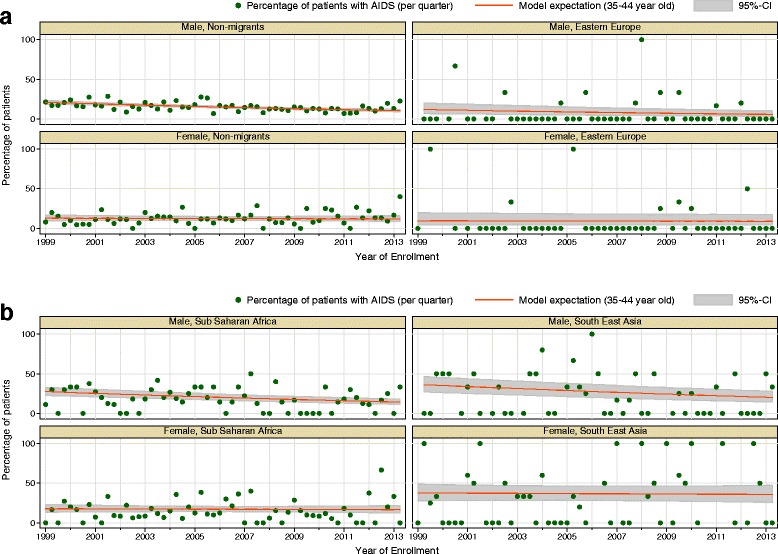
Logistic regression model for time trend of percentage with AIDS at enrolment stratified by sex, 1999–2013; **a** Non-migrants and migrants from Eastern Europe, **b** Migrants from Sub-Saharan Africa and South East Asia; only selected migrant groups shown

Over time, a consistent decrease in percentage of patients enrolling in the cohort with AIDS-diagnosis is seen in male patients of all groups. However, this decreasing time trend is not visible for female patients. (See Table 2 and Fig. 3a and b). For migrants from Eastern Europe the proportion of patients with linkage to care after AIDS-diagnosis is significantly lower (6%) than for non-migrants and all other migrant groups, as not only descriptive but also regression analysis show (OR_EE_ = 0.52, 95%-CI: 0.30 – 0.69; see Table 1 and 2).

### CD4-count

CD4-count at enrolment was available for a total of 9080 of the 9320 patients of the migrant groups of interest. In descriptive analyses, mean CD4-count at enrolment was lower in the Central European, Sub-Saharan African and South East Asian migrants than in non-migrants, while for migrants from Eastern Europe a higher CD4-count is seen (see Table 1). Using 35 to 44-year old, male non-migrants as reference, regression analysis showed a lower mean CD4-count at enrolment for migrants from Sub-Saharan Africa (IRR: 0.72; 95%-CI: 0.64 - 0.82) and South-East Asia (IRR: 0.62, 95%-CI: 0.49 - 0.78) (see Table 3).

**Table 3:** Multivariable negative binomial regression model^a^ for mean CD4-count enrolment into ClinSurv, 1999–2013 (*n* = 9080); significant results in bold

Mean CD4-count (cells/μl) at enrolment	Ratio of mean CD4-count (IRR)	95%-CI	*p*-value
**Region of origin o﻿f migrant group**			
Non-migrants	Ref.	--	--
WE	0.94	0.75–1.16	0.551
CE	0.97	0.83–1.14	0.733
EE	0.84	0.68–1.03	0.092
**SSA**	**0.72**	**0.64–0.82**	**<0.001**
**SEA**	**0.62**	**0.49–0.78**	**<0.001**
**Age Group (in years)**			
**15–24**	**1.37**	**1.28–1.46**	**<0.001**
**25–34**	**1.12**	**1.08–1.17**	**<0.001**
35–44	Ref.	--	--
**45–54**	**0.88**	**0.83–0.92**	**<0.001**
**55+**	**0.78**	**0.73–0.84**	**<0.001**
**Sex**			
Male	Ref.	**--**	
**Female**	**1.07**	**1.02–1.13**	**0.007**
**Time trend (per year) by region of origin, 1999–2013**			
**Non-migrants**	**1.01**	**1,01–1.02**	**<0.001**
WE	1.01	0.98–1.03	0.509
CE	**1.03**	**1.01–1.05**	**0.008**
EE	0.97	0.94–1.00	0.065
SSA	1.01	1.00–1.02	0.139
SEA	1.01	0.99–1.04	0.367
**Mean CD4-count (cells/ul) at enrolment**	**Incidence rate**	**95%-CI**	***p*** **-value**
**(reference group) 2013**	**383**	**365–403**	**<0.001**

^a^Dispersion parameter: Alpha = 0.76 (95%-CI: 0.73–0.78)

**Fig. 4:**
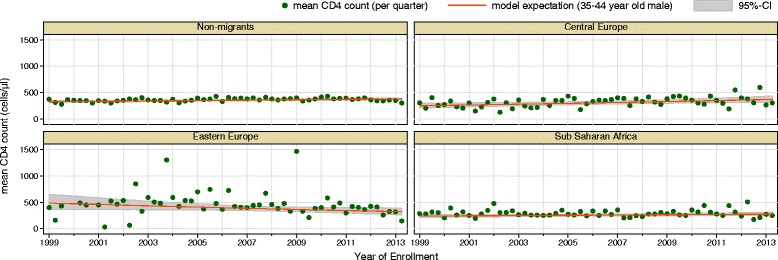
Negative binomial regression model for timely trend in mean CD4 count at enrolment, 1999–2013, only selected migrant groups shown

From 1999 to 2013 mean CD4 count at enrolment increased by 1%/year (95%-CI: 1%–2%) for non-migrants and 3%/year (95%-CI: 1%–5%) for migrants from Central Europe. For all other migrant groups, no significant trend is visible (see Table 3, Fig. 4).

## Discussion

Zoufaly et al. previously used ClinSurv to investigate characteristics of late presentation for HIV care [12]. They showed that migration status constitutes a risk factor for late clinical presentation (AIDS prior to the first visit in a ClinSurv centre or CD4-count lower than 350 cells/ul) to HIV care centres. As migrant groups in Germany are heterogeneous in their formation and underlying risk factors for HIV-infection, it is important that this present study managed to disentangle Zoufaly et al.’s captured differences in regards to different migrant groups. Our results identified, that especially HIV-positive migrants from Sub-Saharan Africa and South-East Asia are linked to specialized care at a later stage of HIV infection than non-migrants. The risk of presentation to HIV-care with AIDS-diagnosis is elevated in these groups compared to non-migrants. For female migrants from South-East Asia, this increased risk is in particular alarming. From 2008 onwards, German treatment guidelines recommend initiation of ART at higher CD4-levels [5, 6] This might be an explanation for the observed overall decrease in the proportion of patients presenting to specialized care with AIDS from 1999 to 2013 in our cohort. Why this decrease is lacking to be observed for female patients of all groups, needs to be further explored, especially since all patient groups irrespective of their sex have found to be linked to care at increasingly higher CD4-counts over time in our study population. Mean CD4-counts of South-East Asia and Sub-Saharan African migrant groups at linkage to care were identified to be lower than for non-migrants and Western European migrants. For migrants from South-East Asia, the mean CD4-counts at first contact to specialized care were found below 300 cells/μl throughout the whole study period, which makes initiation of treatment at below 350 cells/ul according to guidelines challenging.

In line with this, a few very recent studies in Europe captured the decreased CD4-count of certain migrant groups (from the Asian continent and the Sub-Saharan African region) compared to native populations at initiation of ART [15, 16]. Parallel to this, when analysing the presentation of different migrant populations to ART initiation, the German ClinSurv cohort showed similar elevated risks for Sub-Sahara African and South East Asia migrants. We did not investigate, whether as a possible consequence of this presentation to care and ART-initiation after presence of AIDS or with low CD4-counts, further clinical outcomes or accelerated death occured in our cohort.

To get an insight in the population of people living with HIV in Germany at point in time previous to linkage to HIV-care, namely the first HIV-test in Germany, Zoufaly et al. performed additional analysis of German HIV surveillance data in addition to the ClinSurv analysis [12]. It provides case-based data on every new-diagnosed case of HIV through notification by the diagnosing laboratories. However, as HIV surveillance data lacks information on clinical and immunological parameters for many cases and is not linkable to the ClinSurv cohort, this additional method was not applied to our research on different migrant groups. Nevertheless, Zoufaly et al.'s findings showed that migrants also present late for HIV-testing in Germany. This could possibly constitute a contributing factor for later linkage to care of the migrant populations seen in our analysis. In this context, evidence on migrants’ late presentation for HIV-testing in Europe has been extensive in the past [17–20]. Particularly the African migrant population has been found particularly at risk for late HIV-testing [21, 22]. One study found a delayed testing of this migrant group, but observed an uptake of HIV-care similar to non-migrants [23].

Results of participatory research at the Robert Koch Institute among migrants from Sub-Saharan Africa in Germany (MiSSA) show that migrants of this group were often not aware of the anonymous and free HIV-testing offer or assumed that a positive HIV-test will impact their legal status in the country [24]. These together with the fear of stigmatization of HIV-positive migrants in their communities have constituted barriers to HIV testing in African migrants in the past [22, 25–28]. These identified barriers underline our interpretation that migrants from this group may refrain more frequently from HIV testing.

In contrast to other migrant groups, Eastern European migrants present to care at an earlier stage of HIV-infection than non-migrants in terms of AIDS and mean CD4-count. A reason for this could be that a large part of the HIV-infections in the Eastern European migrant population in Germany is acquired via IDU and MSM transmission. For those risk groups HIV-testing is especially promoted in Germany. Other migrant groups, such as South-East Asian migrants and Sub-Sahara African migrants consist of females, predominantly infected by heterosexual transmission – risk groups, who are not especially targeted for HIV-testing. This fact underlines once more the importance of accounting for the different composition of migrant groups in terms of HIV.

According to a recent review on HIV-testing policies in Europe, the German HIV-testing framework, although acknowledging migrants as a vulnerable group for HIV has so far neither issued a tailored recommendation for migrants for HIV-testing within the HIV-testing strategy [29] nor have all of them yet access to HIV-tests in Germany.

RKI conducted first efforts to establish guidelines for a more tailored HIV-testing framework for migrants in Germany in the MiTest-Study between 2014 and 2015 [30]. This mixed-method research included focus group discussions and surveys of cultural mediators, physicians and other staff at HIV-test centres in seven German cities. As a result, useful outreach approaches for certain migrant groups in Germany, such as visiting asylum seeker centres, women’s groups and religious assemblies for HIV testing information could be identified. The study furthermore reports young South-East Asian women as a subgroup of migrants with particularly limited access to HIV-test offers due to their lack of essential knowledge on HIV, which could be an attributable factor explaining our findings.

The legal and political framework for migrants in Germany, the complex composition of the migrant pool in Germany, the lack of more need-adapted testing offers and culture-related factors (such as lack of HIV knowledge and feared stigmatization) nevertheless still form barriers for migrants’ HIV-test seeking uptake, according to the study.

However, when interpreting our results some limitations must be considered.

The majority of migrants recorded in the ClinSurv cohort are covered by statutory health insurance and have regular access to the health care system in Germany. As a consequence, the study population lacks information regarding the proportion of migrant people with AIDS symptoms without any access to the German health care system (for example undocumented migrants). The proportion of AIDS among this group might be higher than observed in our study collective.

Secondly, small sample sizes of certain migrant groups for example the number of enrolled Eastern European migrants in the cohort, make interpretation of the results for these particular subgroup only possible with caution.

Last, no information is available about the arrival date of the migrants in Germany and also no documented information about the first HIV test of the patients. Therefore no conclusions about HIV disease progression since arrival in Germany can be drawn out of the cohort data. As past evidence and current recommendations illustrate, it would be helpful to include information about migrants’ arrival date in Germany into HIV-notification system to close this interpretation gaps in the future [31, 32]).

## Conclusions

In conclusion, we show that particularily migrants from Sub-Saharan Africa and South-East Asia in Germany enter HIV-care at a later stage of HIV infection than non-migrants. This may be a result of late presentation for HIV-testing, as previous research indicates. Collecting information about the arrival date of migrants in Germany in the HIV notification system would help to understand to which extent this problem could be tackled in Germany.

In order to reduce the proportion of late presentation among those migrants groups, the existing participatory approaches for HIV-testing and care should be further strengthened. It is important to disentangle even more the heterogeneous group of migrants, to identify which particular groups still remain unreached by those approaches and identify their specific community organizations in Germany. This opens the door for implementation of knowledge attitude, behaviours and practice studies - similar to the research performed within migrant groups from Sub-Saharan Africa in Germany (MiSSA) - for further migrant groups aiming to develop new tailored strategies, which will improve these groups’ access to HIV-testing and care.

## Abbreviations

95%-CI: 95%-confidence interval
AIDS: Acquired immunodeficiency syndrome
ART: Antiretroviral treatment
CDC: Centers for Disease Control and Prevention
CE: Central Europe
ClinSurv: Clinical Surveillance of HIV Disease
ECDC: European Centre for Disease Prevention and Control
EE: Eastern Europe
HIV: Human immunodeficiency virus
HPC: High prevalence country
IDU: Injecting drug use
IfSG: German Protection against Infection Act
IRR: Incidence rate ratio
MiSSA: Migrants from Sub-Saharan Africa
MSM: Men who have sex with men
OR: Odds ratio
SEA: South-East Asia
SSA: Sub-Saharan Africa
START: Strategic Timing for Anti-Retroviral Treatment
WE: Western Europe
WHO: World Health Organization
